# Effects of Selected Food Additives on the Gut Microbiome and Metabolic Dysfunction-Associated Steatotic Liver Disease (MASLD)

**DOI:** 10.3390/medicina61020192

**Published:** 2025-01-22

**Authors:** Sara Jarmakiewicz-Czaja, Aneta Sokal-Dembowska, Rafał Filip

**Affiliations:** 1 Faculty of Health Sciences and Psychology, University of Rzeszow, 35-959 Rzeszow, Poland; sjczaja@ur.edu.pl (S.J.-C.); asokal@ur.edu.pl (A.S.-D.); 2 Gastroenterology Clinic, Center for Comprehensive Treatment of Inflammatory, Bowel Disease Regional Hospital No. 2 in Rzeszow, 35-301 Rzeszow, Poland; 3 Department of Internal Medicine, Faculty of Medicine, University of Rzeszow, 35-959 Rzeszow, Poland

**Keywords:** colors, emulsifiers, food additives, MASLD, microbiota, preservatives, sweeteners, taste enhancers

## Abstract

The purpose of this article is to present selected food additives as disruptors of normal intestinal homeostasis with a potential impact on the development of metabolic dysfunction-associated steatotic liver disease (MASLD). A comprehensive literature search was conducted in three major electronic databases: PubMed, ScienceDirect, and Google Scholar. MASLD is a prevalent liver condition that is closely related to the global rise in obesity. Its pathogenesis is multifactorial, with genetic, environmental, and metabolic factors playing a key role. The “multiple-hit” hypothesis suggests that a Western-style diet, rich in ultra-processed foods, saturated fats, and food additives, combined with low physical activity, contributes to obesity, which promotes lipid accumulation in the liver. Recent studies underscore the role of impaired intestinal homeostasis in the development of MASLD. Food additives, including preservatives, emulsifiers, and sweeteners, affect gut health and liver function. Selected preservatives inhibit pathogenic microorganisms but disrupt the intestinal microbiota, leading to changes in intestinal permeability and liver dysfunction. Some emulsifiers and thickeners can cause inflammation and alter the gut microbiome, contributing to liver steatosis. Furthermore, the use of sweeteners such as sucralose and aspartame has been linked to changes in liver metabolism and intestinal microbial composition, which in turn promotes metabolic disorders.

## 1. Introduction

Metabolic dysfunction-associated steatotic liver disease (MASLD) was previously referred to as nonalcoholic fatty liver disease (NAFLD). NAFLD was first described by Ludwig et al. in 1980 [1]. In 2020, the term steatohepatitis associated with metabolic dysfunction (MAFLD) was introduced, while in 2023, an international panel of experts proposed a new nomenclature—MASLD [2]. In 2018, the estimated prevalence of NAFLD worldwide was 24% [3]. Currently, is one of the most common liver diseases in the world, and its incidence is increasing every year not only in the adult population but also in children, in parallel with the obesity epidemic [4,5]. MASLD is associated with a greater predisposition to cardiovascular disease, type 2 diabetes, kidney disease, and other diseases [6,7]. The diagnosis of MASLD requires the presence of one or more metabolic risk factors for the disease, i.e., hyperglycemia, excessive body weight, abnormal lipid metabolism, and high blood pressure [8]. It can also predispose people to steatohepatitis associated with metabolic dysfunction (MASH), organ fibrosis, cirrhosis, and hepatocellular carcinoma (HCC) [9]. There are several risk factors for MASLD, but in recent years, researchers have increasingly turned their attention to the disruption of the intestinal microbiota and the disruption of the integrity of the intestinal barrier. The purpose of this article is to present selected food additives as disruptors of normal intestinal homeostasis with a potential impact on the development of MASLD.

## 2. Methods

The data collection process took place from October to December 2024. A comprehensive literature search was conducted in three major electronic databases: PubMed, ScienceDirect, and Google Scholar. The search strategy used a combination of relevant keywords and phrases in two main categories: (i) intervention terms: food additives, colors, emulsifiers, preservatives, sweeteners, or taste enhancers; (ii) condition terms: MASLD and microbiota, MASLD, or microbiota. Articles published in peer-reviewed journals were included if they met the following criteria: (i) written in English, (ii) published between 2019 and 2024, and (iii) directly addressed the relationship of food additives to gut microbiota and MASLD. Publications published prior to 2019 were also reviewed if they were considered crucial to providing basic knowledge. The search for publications was carried out by selecting titles and abstracts, followed by a review of the full text to ensure appropriate methodological quality. The literature was also hand-searched to find other relevant articles. Randomized controlled trials, meta-analyses, systematic reviews, prospective cohort studies, and animal and in vitro studies were analyzed.

## 3. Pathogenesis

The pathogenesis of MASLD is multifactorial and complex. The most commonly indicated link is between genetic, environmental, immunological, and metabolic factors, so the hypothesis of “multiple hits” has been proposed [10,11].

One of the main modulators of pathogenesis is the adherence to a Western-type diet, containing ultra-processed foods, high in saturated and trans fatty acids and food additives, and with a lack or low levels of physical activity. This type of lifestyle predisposes people to insulin resistance, being overweight, or obesity. This, in turn, affects organokines (gut cytokines, osteokines, adipokines, and myokines) and, more specifically, the amount of their secretion. An example is the reduction in Nrg4 (neuregulin 4), which is secreted by brown adipose tissue, which can consequently cause disturbances in liver metabolic homeostasis. Another example is cell communication network factor 4, which is secreted by adipose tissue, altering the action of, among other things, insulin [12]. Excess body weight predisposes people to the hepatic accumulation of triglycerides from non-esterified fatty acids secreted from adipose tissue [13]. MASLD also has an increased rate of de novo hepatic lipogenesis, which accelerates lipid accumulation in the organ [14]. Other risk factors are the gene polymorphisms present, e.g., patatin-like phospholipase domain-containing protein 3, membrane-bound O-acyltransferase domain-containing protein 7, or transmembrane 6 superfamily 2. Some also show an increased predisposition to liver fibrosis and the appearance of HCC [15,16,17,18]. Furthermore, gut microbiota metabolites can affect liver lipogenesis. One of them is short-chain fatty acids (SCFAs), which are formed from dietary fiber; more specifically, propionic acid is formed, which can predispose people to gluconeogenesis and adipogenesis, which have been shown to have an effect on the development of MASLD [19]. Another metabolite is bile acids, whose primary role is the digestion of lipids, but they have also been shown to help maintain the normal homeostasis of the intestinal microbiota [20]. A different example is choline and its metabolites, mainly trimethylamine (TMA). TMA is metabolized by the gut microbiota, absorbed through the intestines, and transported to the liver. Therefore, diets deficient in choline affect the occurrence of intestinal barrier disorders, which predispose people to the accumulation of lipids in the liver and the onset of organ steatosis [21]. Additionally, the sheer disturbance in the quantity and quality of the gut microbial composition can influence the onset of MASLD. Proteobacteria are observed more frequently in the presence of hepatic steatosis [22]. Boursier et al., in their work, observed that *Bacteroides* can be associated with nonalcoholic steatohepatitis, while *Ruminococcus* can be associated with the progression of organ fibrosis [23]. In another paper, Zhang et al., after observing animal models fed a high-fat diet, found that there was an increase in *Mucispirillum and Desulfovibrio,* among others, while there was a decrease in *Bacteroides* and *Bifidobacterium*, which was associated with the development and progression of MASLD to the onset of HCC [24]. However, there is currently a lack of long-term studies identifying a clear link between the gut microbiota and the occurrence of MASLD.

**Figure 1:**
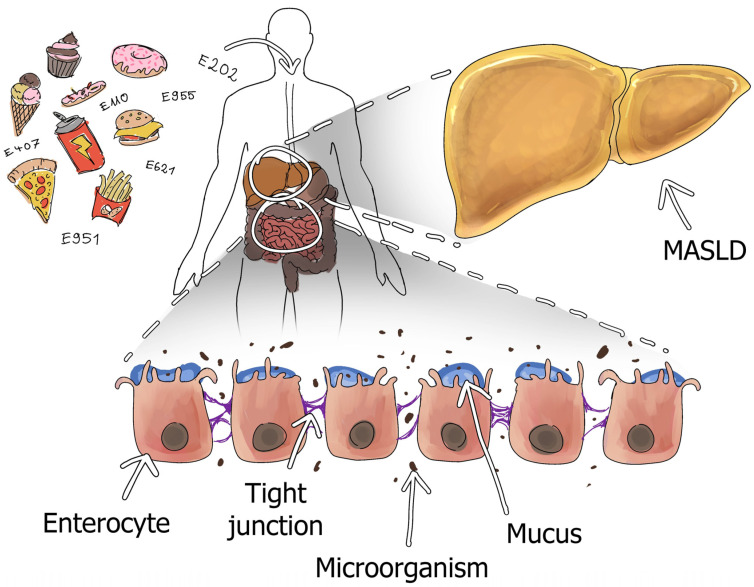
Potential influence of selected food additives on the association with intestinal homeostasis disorders and the occurrence of MASLD. Selected food additives can adversely affect the maintenance of intestinal homeostasis by reducing normal tight junction function, decreasing mucus, and altering the composition and quantity of certain intestinal microorganisms. And these changes affect the liver, leading to an increased risk of MASLD.

The mechanism of the MASLD formation process is complex and multifactorial. An influx of released free fatty acids (FFAs) from adipose tissue into the liver, along with the body’s hyperinsulinemia, causes an imbalance between hepatic lipid absorption and excretion. Donnelly et al. indicate that approximately 59% of hepatic lipids from MASLD come from FFA, about 26% from the de novo lipogenesis pathway, and only about 15% from the diet [14]. Due to the accumulation of lipotoxic fatty acid β-oxidation intermediates, mitochondria malfunction, which, in turn, reinforces the lack of fatty acid burning efficiency [25,26]. Mitochondria produce 90% of cellular reactive oxygen species (ROS); with uncontrolled mitochondrial oxidative stress, there is oxidative damage to hepatocytes [27]. Endoplasmic reticulum stress triggers inflammatory cascades by increasing the activity of nuclear factor κB, c-Jun N-terminal kinase, and others, which can regulate inflammatory macrophage activation [28]. Activated macrophages after organ damage secrete pro-inflammatory cytokines, such as interleukin-6 (IL6), human tumor necrosis factor-α (TNFα), and interleukin-1β (IL1β), and so inflammation increases [29]. Macrophages and Kupffer cells (KCs) are also involved in the inflammatory process. Activated KCs, through liver injury, like macrophages, promote inflammatory reactions occurring in the organ, which causes the activation of hepatic stellate cells (HSCs) [30,31]. These, in turn, are a key element for liver fibrosis, which in advanced cases can lead to organ cirrhosis [32,33]. The simplified connections between the gut and liver are shown in Figure 1.

## 4. Nutritional Models Used in the Management of MASLD

As has been demonstrated, diet is one of the most significant modifiable factors in lifestyle that influence the diversity of the host microbiome [34]. There is clear evidence that the proper state of the microbiota plays a crucial role in the pathogenesis of numerous diseases, as well as in their prevention and management [35,36]. Dietary models based on a high supply of dietary fiber are an important part of the prevention of intestinal diseases [35] and diet therapy for chronic liver diseases, including MASLD [37]. An analysis of the association between dietary fiber intake and MASLD was evaluated in the NHANES study among 5935 participants, which confirmed that there is an inverse relationship between dietary fiber intake and changes in liver steatosis [38].

Dietary fiber modulates the gut microbiota, as it is one of the main substrates used by gut microbes. The fermentation of dietary fiber and resistant starch in the large intestine results in the production of SCFAs, including acetate, butyrate, and propionate [35]. These substances demonstrate anti-inflammatory, immunomodulatory effects and improve intestinal barrier function [39,40].

Fiber also acts as a prebiotic, promoting the growth of beneficial gut bacteria such as *Bifidobacterium*, *Lactobacillus*, *Faecalibacterium*, *Ruminococcus*, *Akkermania,* or *Roseburia*, which contribute to improved intestinal health and the overall health of the body [41,42]. Regular consumption of fiber can improve the composition of the microbiota, which can have a long-term impact on reducing the risk of chronic diseases such as obesity, type 2 diabetes, and heart disease, which are often associated with MASLD [43].

New therapeutic approaches to the nutritional management of MASLD are constantly being sought. Hansen et al. showed that a low-carbohydrate, high-fat diet (50–60% fat, less than 20% carbohydrate, and 25–30% protein) can result in significant improvements in glycemic and weight control compared to a high-carbohydrate, low-fat diet (50–60% carbohydrate, 20–30% fat, and 20–25% protein) [44]. Similarly, Chen et al. reported improvements in body composition parameters and alanine transaminase (ALT), aspartate aminotransferase (AST), uric acid, and insulin levels in patients with MASLD following a low-carbohydrate, high-fiber diet combined with nutritional education [45]. Cunha et al. reported better weight reduction accompanied by substantial decreases in visceral adipose tissue and liver fat fractions in comparison with the standard diet [46].

Nevertheless, the low-calorie Mediterranean diet (MED) remains the most frequently recommended dietary intervention for managing MASLD [47,48]. Montemayor et al. conducted a multicenter (Mallorca and Navarra, Spain) prospective randomized trial to test the effect of the MED diet in patients with MASLD and metabolic syndrome. A six-month follow-up showed that adherence to the diet led to lower levels of parameters, such as body mass index (BMI), body weight, waist circumference, and intrahepatic fat content, and lower levels of blood pressure (systolic blood pressure and diastolic blood pressure) [49]. However, preliminary results of a multicenter RCT (randomized controlled trial) conducted by Rosi et al. showed no difference between the use of an MED diet and a low-fat diet in counteracting obesity in children and adolescents [50]. However, it should be borne in mind that the use of dietary questionnaires to assess dietary habits may be subject to measurement error.

Cheng et al. showed that dietary intervention combined with physical activity positively affects the stability of the ecosystem interaction network, thereby improving host metabolism [51]. An interesting intervention was carried out by Chooi et al. The researchers included supplementation with pentadecanoic acid (C15:0), which naturally occurs in milk fat and ruminant meat, in the diets of women with MASLD. The use of a diet rich in dietary fiber and unsaturated fatty acids in combination with C15:0 led to a reduction in low-density lipoprotein (LDL) cholesterol levels and an increase in the abundance of *Bifidobacterium adolescentis* [52]. In the Gómez-Pérez study, an MED diet combined with physical activity for 12 months in patients with clinically suspected MASLD and MASH resulted in improved gut microbiota composition, which was associated with changes in MASLD/MASH biochemical indices (non-suspected fibrosis and indeterminate or suspected fibrosis). There was an increase in the genus *Coprococcus* and *Lachnospira*, as well as *Oscillospira*, and a decrease in *Proteobacteria* and its family *Enterobacteriaceaee* [53].

An analysis of recent data by Bialczyk et al. showed that the introduction of probiotic therapy in patients with MASLD can favorably affect liver enzyme levels, improving insulin sensitivity, lipid profile parameters, and BMI. Probiotic supplementation can reduce inflammatory markers such as IL-6, c-reactive protein (CRP), and TNF-α, and, when combined with prebiotics, can also improve histological markers [54]. *Lactobacillus* may be beneficial in alleviating MASLD through their effects on various tissues and organs in the body, and their effectiveness may vary depending on the strain and the patient’s current condition. *Lactobacillus* can restore intestinal homeostasis by modulating Mucin2 and intestinal tight junctions [55]. In patients with MASLD, the use of multi-strain probiotic therapy (six different species of *Lactobacillus* and *Bifidobacterium*) at a concentration of 30 billion CFU for 6 months did not significantly affect the degree of steatosis/fibrosis or improve laboratory parameters. However, due to the significant reduction in the expression of CD8+ T lymphocytes and zonula occludens-1 (ZO-1) in the placebo group, the authors suggest that probiotics may play a role in stabilizing the immune function of the mucosa and also prevent intestinal permeability in MASLD patients [56]. An interesting observation was made by Xue et al., who performed fecal microbiota transplantation (FMT) on patients with MASLD from healthy donors. Patients who received FMT showed a reduction in fat accumulation in the liver by improving intestinal microbiota dysbiosis, thereby reducing hepatic steatosis, especially in patients suffering from obesity. The control group who received probiotic therapy (*Bifidobacterium viable* and *Lactobacillus acidophilus* capsules) showed no differences in blood lipid levels, liver function, and fat suppression before treatment [57].

**Figure 2:**
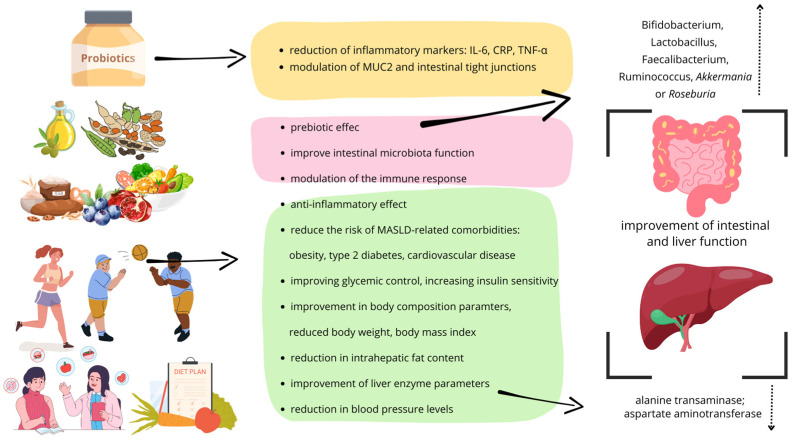
The benefits of a diet rich in dietary fiber and unsaturated fatty acids, combined with exercise in the context of improving intestinal and liver function and preventing metabolic disorders associated with MASLD. Il-6—interleukin 6; CRP—c-reactive protein; TNF-α—tumor necrosis factor alpha; MUC2—Mucin2.

According to the available data, a low-processed diet, rich in dietary fiber, focusing on the intake of whole grain products, vegetables, fruits, and unsaturated fatty acids through the modulation of the intestinal microbiota, promotes intestinal health and may be key in the prevention of MASLD (Figure 2).

## 5. Food Additives in Association with the Gut Microbiome and MASLD

### 5.1. Colors (E100–E199)

It is widely accepted that food colors are among the most toxic food additives used in the food industry, with those belonging to the ’azo’ group being considered to be the most genotoxic. Tatrazine (TS) (E102) is an artificial dye that contains an azo group and is soluble in water [58,59]. It is a compound commonly used in the food industry. Due to its yellow color, it is often added to yellow cheeses, sauces, jellies, chewing gums, fish products, flavored wines, and other drinks, such as soft drinks and sports drinks. It is also an additive to vegetable and fruit products—both canned and bottled [58,60]. The acceptable daily intake (ADI) level according to the Food and Drug Administration was recognized at 5 mg/kg bw in 2011, while the European Food Safety Authority approved it at 7.5 mg/kg bw [60]. TS, to a small extent, can be reduced by various bacterial taxa [61]. Azoreduction of TS in the gut leads to the formation of sulfanilic acid and 4-amino-3-carboxy-5-hydroxy-1-(4-sulfophenyl)pyrazole (SCAP) [62]. TS metabolites are considered potentially hazardous to health, especially for children who consume significant amounts of colored foods and soft drinks [63]. Nitrogen dyes account for 10–22% of the maximum ADI in beverages [64]. Subsequently, toxic concentrations of SCAP can occur in the gut when TS is consumed at the limit of the recommended daily dose [62].

A study by Wu et al. showed that the ingestion of TS (1.4, 5.5 and 10 mg/kg/day) could cause severe histopathological and cellular changes in the intestines and liver of goldfish. Some epithelial cells became vacuolized and the intestinal villi was ruptured. In addition, TS supply induced oxidative stress (proportional dose-dependent increase in malondialdehyde (MDA)) and led to changes in the intestinal microbiota, an increase in *Actinobacteriota* and *Proteobacteria*, and a significant decrease in *Planctomycetota* and *Fusobacteriota*, as well as the bacteria responsible for SCFA production (*Bacteroides* and *Clostridium_sensu_stricto_1*) [65]. In a subsequent study, Wu et al. reached similar conclusions. TS supply was associated with changes in the intestinal microbiota and the development of inflammation, which was associated with the up-regulation of pro-inflammatory cytokines (IL1 and IL6), lysozymes (lyz), β-defensin 3 (defb3), and complement component 3 (c3) [66].

In a study by El-Desoky et al., the administration of TS at a dose of 7.5 mg/kg bw for 50 days resulted in increased liver function enzymes (aspartate aminotransferase (AST), alanine aminotransferase (ALT), gamma-glutamyl transferase, and alkaline phosphatase), bilirubin levels, abnormal lipid profile, and serum glucose. There was a decrease in body weight with an increase in liver weight, indicating its toxic effects. An increase in protein kinase C (PKC) isoforms was indicative of ROS generation, and alpha-fetoprotein was indicative of liver failure [67].

As with TS, Allura Red (AR) (E129) can be metabolized by an intestinal bacterium through nitrogen reduction, including *O. splanchnicus* and *P. vulgatus*. This results in the formation of two compounds, creisin-4-sulfonic acid and 1-amino-2-naphthol-6-sulfonic acid [61,68]. Hofseth et al. believe that the association of AR with inflammation, DNA damage, and the concomitant disruption of the microbiome is notable [68]. Kwon et al. observed that chronic, long-term exposure to AR promotes experimental colitis via serotonin in the colon in a pathway, whether dependent on or independent of the gut microbiota, in mice [69]. He et al. noted that the risk of developing colitis was increased in mice expressing the increased expression of IL-23, leading to the increased generation of activated CD4 T cells that expressed interferon-γ. In turn, the induction of colitis was dependent on the commensal microbiota, promoting AR azo reduction and the production of the metabolite 1-amino-2-naphthol-6-sulfonate sodium [70].

The administration of AR to albino rats for 4 weeks at a dose of 7 mg/kg bw resulted in an increase in biochemical markers of liver function (ALT and AST) and MDA levels and a decrease in serum antioxidants. There were changes in the histological structure with a decrease in Bcl2 expression and an increase in cytochrome c oxidase subunit II expression [71].

Sunset yellow (SY) (E110), also commonly used in the food industry, is added to foods such as desserts, chips, cookies, ice cream, and soft drinks and also to pharmaceutical products and drugs and syrups for children [72]. Due to its potential mutagenic and carcinogenic effects, SY has been banned for use in Norway and Finland [73]. The ADI of SY is 4 mg/kg/bw [74].

According to Sensoy et al., SY can affect the intestinal epithelium, inducing changes in intestinal secretion. SY has also been shown to interfere with intestinal signaling interactions by exerting antagonistic effects on the glucagon-like peptide-1 (GLP-1) receptor, a peptide hormone [72]. In the study by Zahran et al., SY administration at a dose of 6.17 mg/kg (equivalent to human ADI of 1 mg/kg) for 12 weeks increased serum LPD and altered the intestinal microbiome, leading to the disruption of intestinal integrity by altering the jejunal E-cadherin/β-catenin adhesion junction complex and decreasing cloverleaf factor (TFF)-3. SY decreased the abundance of beneficial taxa, including Treponema 2 and *Anaerobiospirillum*, while increasing the abundance of potentially pathogenic microorganisms *Prevotella* and *Oribacterium* [75].

Abdelhamid et al. have shown that SY can induce a number of adverse structural and biochemical changes in the liver [76]. In the previously mentioned study, Khayytat et al. observed similar changes when SY was administered at a dose of 2.5 mg/kg body weight as when AR was used. However, an additional genotoxic effect was observed for SY, which was not detected for AR [71]. Huessein et al. reported that the long-term oral administration of SY above ADI is hepatotoxic and has negative effects on immunity. However, the authors noted an increase in ALT and AST and, even at low doses of SY, an increase in the mRNA expression of proapoptotic protein [77].

Data on the effects of food dyes on the gut microbiota and liver function come mainly from studies on animal models. However, the results of these studies indicate that these substances can cause negative health effects in humans as well. Dyes commonly used in the industry can change the composition of the intestinal microbiota, leading to structural changes in the intestines, with the liver disrupting their function. Thus, they lead to the development of inflammation and a decrease in antioxidants in the body.

### 5.2. Preservatives (E200–299)

This section concerns a group of food additives labeled E200 to E299. They have their use in cured meats, sauces, marinades, and processed foods [78]. Some preservatives have been shown to inhibit the growth of beneficial microorganisms, thereby disrupting intestinal microbial homeostasis and causing predisposition to inflammation [79]. Another example of substances used in the food industry with a preservative effect are sulfites. Their main function is to inhibit the growth of pathogenic microorganisms. However, they can exhibit a number of other undesirable functions. Irwin et al. point out that sulfites can potentially lead to cell damage reactions; additionally, due to their bacteriostatic and bactericidal effects, they can potentially alter the oral and intestinal microbiome [80,81]. In a study by Nagpal et al., in animal models, the authors observed a qualitative and quantitative change in the composition of the intestinal microbiota after treatment with potassium sorbate (E202), benzoic acid (E210), or sodium nitrate (E251). Changes related to intestinal epithelial permeability and the expression of markers of intestinal tight junctions were also demonstrated [82]. In addition to their indirect effects on the liver through the modulation of the gut microbiota, preservatives added for food preservation can also have direct effects. Hrncir et al. studied liver function after exposure to fructose and preservatives such as sodium benzoate, sodium nitrite, and potassium sorbate. They noted that preservatives can amplify the adverse effects of fructose on liver function and lipid metabolism. Such synergistic adverse effects have also been observed to increase intestinal permeability [83]. Crowe et al. examined the effects of sodium nitrite found in sausages on the colorectal cancer status in mice. They showed that the consumption of meat products with sodium nitrite added was associated with intestinal dysbiosis and higher lipid peroxidation [84]. On the contrary, Van Hecke et al. show that the consumption of peated beef compared to fresh beef alters the composition of the intestinal microbiota in animal models with a higher relative abundance of *Ruminococcaceae* [85]. Preservatives can have an indirect effect on the onset of homeostatic disorders leading to MASLD, but some studies indicate a direct effect by disrupting normal organ function.

### 5.3. Emulsifiers, Thickeners, Stabilizers (E400–499)

Food additives in the group of emulsifiers, thickeners, and stabilizers are designated as E400-E499. An example of a food additive with thickening and emulsifying properties is carrageenan (E407). It is extracted from the cell walls of red seaweed. It is often an ingredient in fat-reduced food products, for example dairy products, cured meats, dietary supplements, jams, jellies, powdered products, instant drinks, and sauces [86]. Borsani et al. in their paper presented that carrageenan can induce inflammation, predisposing people to the onset of exacerbation in ulcerative colitis (UC) and thus disrupting intestinal homeostasis and the integrity of the intestinal barrier, and can cause an increased risk of bacterial translocation, which would also have consequences for the liver [87]. Ariffin et al. studied the effects of carrageenan on intestinal and hepatic cell lines. They noted that the acid hydrolysis products of k-carrageenin could exhibit cytotoxic effects on both cell lines, while unintegrated carrageenin did not show such properties [88]. In a review article, Liu et al. conclude that the long-term consumption of carrageenan may be associated with inflammation in the gut and changes in the composition of the gut microbiota [89]. Furthermore, Naimi et al. indicate that carrageenan may have adverse effects on the intestinal epithelium and the composition of the intestinal microbiota and may show increased expression of pro-inflammatory molecules [90]. Through this type of interaction, it can potentially cause inflammation that is histopathologically similar to inflammatory bowel disease [91]. All of these disorders can trigger further consequences in the pathogenesis of liver disease [92,93].

Polysorbate 80 (P-80), labeled E433 on the list of food additives, is used as an emulsion stabilizer. It is added primarily during the production of sauces, ice cream, and confectionery products [94]. P-80 or carboxymethylcellulose (CMC, E-466) can cause a predisposition to type 1 diabetes, cardiovascular disease, intestinal disease, and metabolic syndrome [95,96,97,98]. In the case of the direct effects of emulsifiers on the liver, Vilas-Boas et al. describe that there was a predisposition to liver toxicity with formulations in which P-80 was added, which may be the reason for increased membrane permeability [99]. Lv et al., in their study, observed that P-80 can stimulate colitis synergistically with a high-fat diet, affect weight gain, and predispose people to changes in their bile acid profile [100]. In another study in animal models, Singh et al. indicate that P-80-fed mice showed an association with faster fat growth. They also observed elevated parameters indicative of metabolic syndrome and low-grade inflammation, which can lead to MASLD. The authors showed the appearance of abnormalities in the gut microbiota in mice fed P-80 [101]. On the other hand, a direct link to intestinal dysbiosis and the occurrence of MASLD may be caused by the incorrect detection of receptor containing protein 6 (NLRP6) and receptor containing protein 3 (NLRP3) inflammasomes [102].

#### Other Substances with a Thickening Effect Using Maltodextrin as an Example

Although not classified as an emulsifier, maltodextrin (MDX) exhibits thickening properties in starchy products and is widely used as a food additive. Nickerson et al., in their work, indicate that up to 60% of packaged food products may contain MDX or modified starch in their composition [103]. Arnold et al. describe that MDX consumption may predispose people to low-grade inflammation and may be a risk factor for IBD; it may also increase the risk of metabolic disease, which is also associated with MASLD [104]. In another study in animal models, Singh et al. showed that in mouse pups fed a predominantly MDX mixture, it could induce intestinal damage similar to necrotizing enterocolitis (NEC). The consequences included bacterial translocation and the altered functioning of tight junction (TJ) proteins. The factor leading to intestinal damage altered homeostasis between pro-inflammatory cytokines and anti-inflammatory cytokines [105]. In another study, Zangara et al. reached similar conclusions. In addition to increasing susceptibility to colitis in genetically susceptible individuals, it can also alter intestinal mucus production [106]. Almutairi et al. conducted a systematic review of randomized placebo trials in which MDX was used as a placebo. They concluded that because MDX can induce modifications in the gut microbiota and immune factors, it should not be used as a placebo in clinical trials [107]. All of these impacts on the gut microbiota and the disruption of gut barrier integrity could also potentially affect the liver and the appearance of MASLD. However, in many studies on liver effects, MDX is used as a placebo or with other prebiotic substances, and so further studies are needed to determine the actual effects of MDX on the occurrence of MASLD.

### 5.4. Taste Enhancers (E600–699)

The group of food additives known as flavor enhancers is classified in the list from E600 to E699. These are substances added to foods to increase the intensity of certain flavor characteristics and aromas. The flavor enhancers most commonly studied are monoammonium glutamate (MAG) (E624) and monosodium glutamate (MSG) (E621). They are found mainly in powdered soups and sauces, salty snacks, seasoning mixes, stock cubes, and fast food dishes [108,109]. Ahangari et al., in their review article, indicate that the long-term intake of MSG can cause changes in the gut microbiota and have effects on liver metabolism and hepatocyte damage [110]. Nahok et al., in a study using animal models, showed that the metabolic changes that occurred after MSG ingestion are related to gluconeogenesis and branched-chain amino acid metabolism with concomitant intestinal dysbiosis [111]. In another study, the authors indicate that the addition of MSG to the diet causes a reduction in *Akkermansia muciniphila*, which, among other things, produces mucin [112]. In a study by Coelho et al., the researchers observed that in mice with prevalent obesity with MSG in the diet, there is a premature induction of fat accumulation in the liver, predisposing them to the induction of MASLD and subsequent disease progression in the form of hepatitis [113]. MSG causes oxidative stress, which can also contribute to liver damage and changes in liver fat metabolism [114,115]. Olowofolahan et al., after orally administering MSG in various doses to rats, observed that MSG in low doses is tolerated by the animals, while in high doses, it causes cytotoxicity by opening the hepatic mitochondrial permeability transition pore; a similar trend occurred for lipid peroxidation, among other things [116]. Obesity-induced hepatic steatosis while consuming MSG may predispose to the infiltration of lymphocytes, macrophages, and eosinophils, increasing inflammation, which can then cause a predisposition to HCC [117]. Flavor enhancers can have a negative impact on the gut microbiota, and chronic and excessive consumption of these food additives can predispose people to oxidative stress and cause damage to hepatocytes, leading to altered organ function.

### 5.5. Sweeteners (E900–999)

Sweeteners are substances used to add a sweet taste to food products. In this capacity, they function as substitutes for sucrose and other naturally occurring saccharides. Sucralose (E955) is one of the most prevalent sweeteners in the industry. Sucralose is a non-nutritive sweetener, 600 times sweeter than sucrose. For years, it has been recommended to patients suffering from diabetes and obesity. Sucralose is poorly absorbed and enters the lower gastrointestinal tract practically unchanged, where it can potentially alter the composition of the microbiota [118,119]. In the 1990s, it was considered safe by the Food and Agriculture Organization of the United Nations and World Health Organization, and its ADI was set at 15 mg/kg body weight (bw) [118]. Sucralose is often added to foods and beverages, including products designed for patients with diabetes or people who want to reduce energy consumption in their diet [120]. It is also used in the production of alcoholic and non-alcoholic beverages, dairy drinks, chewing gums, ice cream, jams, and jellies [121]. However, in 2023, the WHO has published new guidelines on non-sugar sweeteners (NSS), which advise against the use of NSS for weight management or to reduce the risk of noncommunicable diseases [122]. Feng et al. highlighted the important issue of the influence of other factors, both external and internal, that can affect the microbiome with NSS exposure, such as environmental factors, diet, and stress [123].

According to Shiffman et al., the amount of sucralose 6-acetate in a single daily sucralose-sweetened beverage can far exceed the toxicological risk threshold for genotoxicity (TTC genotox) of 0.15 µg/person/day. Sucralose 6-acetate was shown to significantly increase the expression of genes related to inflammation, oxidative stress, and cancer, with the highest expression observed for the metallothionein 1 G gene (MT1G) [124].

Bian et al. demonstrated that sucralose has the ability to influence the composition of the gut mycobiome and modify its metabolic functions. A six-month supply of sucralose was administered to mice, resulting in alterations in hepatic gene expression (matrix metalloproteinase 2 and inducible nitric oxide synthase (iNOS)) [125]. Sucralose consumption significantly increased the abundance of the intestinal genera *Bacteroides* and *Clostridioides*, which are responsible for the production of deoxycholic acid, in a study of MISICG models, and its increase has been linked to the development of MASLD [126].

The role of gut bacteria in the biotransformation of bile acids is also important. By cleaving amino acid residues, gut bacteria can lead to the uncoupling of taurine/glycine-conjugated bile acids. In a study by Chi et al., sucralose was found to have a reducing effect on the abundance of bacteria associated with the metabolism of bile acids. Furthermore, sucralose administration was found to result in reduced levels of hepatic farnesoid X receptor activation, which was associated with an increase in intrahepatic cholesterol levels [127].

A randomized case–control study by Suez et al. showed that sucralose, at a dose below the ADI, impaired glycemic responses in healthy subjects over two weeks of administration. The increase in glycaemia was accompanied by an increase in plasma trichloroacetic acid and changes in the oral microbiome in the relative abundance of six Streptococcus species in the sucralose group [128]. Different results were obtained by Orku et al. They showed that the regular consumption of water sweetened with sweeteners, including saccharin, in doses similar to those consumed in everyday life had no significant effect on glycemic response, insulin sensitivity, GLP-1 release, and body weight in healthy subjects [129]. Moreover, of all the substances tested (sucralose, aspartame, saccharin, and stevia), dietary supplementation with sucralose had the greatest impact on the functional potential of the human microbiome in an NNS-specific manner [128]. However, the results of a randomized, double-blind, cross-over clinical trial showed that the oral consumption of sweetened beverages at a dose of 136 mg/day had no measurable effect on gut microbiota in healthy participants [130].

Aspartame (E951) is a sweetener composed of two naturally occurring amino acids, L-phenylalanine and L-aspartic acid. It is about 200 times sweeter than sucrose, and the ADI is 40 mg/kg bw [131]. Aspartame is used in the food and pharmaceutical industries and is added to chewing gum, ice cream, dairy products, cough drops, and chewable vitamins [132]. After absorption in the intestinal lumen, aspartame is hydrolyzed to phenylalanine (50%), aspartic acid (40%), and methanol (10%) [133].

Finamor et al. showed that aspartame can negatively affect liver health. Feeding aspartame to mice at a dose of 80 mg/kg for 12 weeks led to increased levels of liver enzymes ALT and AST and liver fibrosis. Increases in profibrotic markers were observed, including transforming growth factor β 1, collagen type I alpha 1, and alpha-smooth muscle actin. Aspartame decreased the activation of erythroid nuclear factor 2-related factor 2 (Nrf2) and increased lipid peroxidation, thereby affecting the activation of NLRP3 [134]. The initiation of the NLRP3 inflammasome has been linked to liver cancer, particularly HCC [135]. Aspartame also reduced levels of peroxisome proliferator-activated receptor gamma coactivator 1 alpha (PGC-1α), and its deficiency may be responsible for changes in the serum lipid profile, as well as lipid accumulation and impaired hepatic gluconeogenesis. Palmnäs et al. demonstrated that the administration of aspartame at a dose of 5–7 mg/kg/d, via drinking water over a period of 8 weeks, was associated with an increase in propionate, a substrate with a high gluconeogenic potential. The increase in propionate, in conjunction with the increase in fasting glucose levels, has the potential to impair insulin-stimulated glucose disposal on both standard and low-fat diets, irrespective of body composition. Furthermore, aspartame intake was found to be associated with an increase in the total number of *Enterobacteriaceae* and *Clostridium leptum* bacteria [136]. In another study, Finamor et al. showed that chronic aspartame administration can lead to hepatic glutathione depletion, which is associated with reduced glutamate cysteine ligase and cysteine levels. Moreover, aspartame induced a blockade of the trans-sulfuration pathway at two steps, namely methionine adenosyltransferase and cystathionine γ-lyase [137].

In the previously cited double-blind study conducted by Ahmad et al., an evaluation of the effects of the oral consumption of aspartame-sweetened beverages at a dose of 425 mg/day showed no effect on the gut microbiota in healthy participants [130]. A randomized, case–control study by Tey et al. demonstrated that the consumption of artificially and naturally sweetened non-calorie beverages, including aspartame, had minimal effects on postprandial glucose and insulin levels in comparison to sucrose-sweetened beverages [138]. Interestingly, changes in insulin sensitivity caused by aspartame supply have been linked to the development of carcinogenesis [135]. Orku et al. [129] and Suez et al. reported comparable outcomes on the impact on the glycemic response. However, the ingestion of sweeteners, including aspartame, has been shown to induce substantial alterations in the composition of the fecal and oral microbiome, as well as the plasma metabolome [128].

Saccharin (E954) is a commonly used sweetener in the food industry, and the ADI is 5 mg/kg bw [131]. It is added to a wide range of foods, from dairy drinks to unripened cheese, jams, confectionery, and even breakfast cereals [139]. Saccharin is not metabolized in the body, but can pass through the placenta and breast milk, so it is not recommended for pregnant or breastfeeding women [131,140]. There is evidence that saccharin can be a metabolic disruptor and can alter the composition of the gut microbiome in offspring [140].

The administration of saccharin in drinking water at a concentration of 0.3 mg/mL over a period of six months resulted in a substantial increase in iNOS and TNF-α levels in the liver of mice. This increase was concomitant with the onset of inflammation and the disruption of the microbiome. The composition of the gut microbiota and the metabolome exhibited alterations. The study noted an increase in bacteria associated with inflammation, including *Corynebacterium, Turicibacter,* and *Roseburia* [141]. Serrano et al. conducted a double-blind, placebo-controlled, parallel study with healthy men and women, as well as an animal model. The study found no effect of saccharin supply on glucose tolerance or gut microbiota composition in humans or mice. However, it should be noted that the study evaluated the effects of short-term saccharin consumption in the maximum permitted amounts [142]. Similarly, Orku et al. did not show an effect of saccharin on glycemic response or insulin sensitivity in healthy subjects [129]. Suez et al. obtained divergent results. They reported a reduced relative abundance of *Fusobacterium* in the oral mycobacterium after saccharin ingestion and an impaired glycemic response during a short-term (two week) supply of saccharin [128]. The recommendation to use sweeteners as substitutes for sugars continues to appear in recommendations, including for people suffering from obesity. Based on the available research results, it is worth noting that the consumption of sweeteners instead of sugar should not be a long-term change, but only a temporary one during a modification of eating habits. 

Table 1 presents the results of studies on the effects of food additives on gut microbiota homeostasis and MASLD discussed in this review.

## 6. Limitations

A limitation is the lack of animal and human studies examining the effects of the food additives analyzed in the publication, their impact on the gut microbiota, and their association with the occurrence of MASLD. There is also a lack of long-term studies on these associations; moreover, some of the mechanisms of association are still poorly understood, and so, in the future, it would be worthwhile to expand research to include other food additives as well.

## 7. Conclusions

Foods rich in food additives, thanks to their properties, are particularly attractive to consumers, especially children and adolescents. Some food additives including emulsifiers, preservatives, flavor enhancers, dyes, or artificial sweeteners may predispose people to dysfunction in the integrity of the intestinal barrier and may affect the composition of the intestinal microbiota, leading to inflammation. These mechanisms can lead to oxidative stress that is not sufficiently compensated for, predisposing the individual to impaired lipid metabolism in the liver. However, further research is still needed to fully elucidate the extent of the health effects of certain food additives and to understand the mechanisms by which they affect the intestinal microbiota and the pathogenesis of MASLD.

Given the increasing incidence of obesity and MASLD, there is a need to effectively control the intake of these substances, particularly among young people, and to further restrict their use in food production. It seems that the recommendations for acceptable intake should be reviewed as well. 

## Figures and Tables

**Table 1:** The results of studies on the effects of food additives on gut microbiota homeostasis and MASLD discussed in this review.

Food Additive Example	Acceptable Daily Intake (ADI) [143]	Potential Impact on Gut Microbiota Disorders in Association with MASLD
Tatrazine (E102)	7.5 mg/kg body weight	Histopathological and cellular changes in the gut and liver [86]; Increase in oxidative stress [86]; Changes in the composition of the intestinal microbiota [66,86]; Liver dysfunction (increased liver enzymes) [67]; Abnormal lipid profile and increased serum glucose levels [67]; Up-regulation of pro-inflammatory cytokines [66]; Increased ROS production [67]; Increase in alpha-fetoprotein in serum [67].
Allura Red (E129)	7 mg/kg body weight	Increased inflammation, disruption of the intestinal microbiota [68]; Liver dysfunction (increase in liver function enzymes) [71]; Increase in serum MDA and NO levels [71]; Decrease in serum antioxidants [71]; Changes in histological structure (disorganization of hepatic strands, necrotic and hydropic degeneration of hepatic cells) [71]; Decreased Bcl2 expression and increased COX2 expression [71].
Sunset Yellow (E110)	4 mg/kg body weight	Disruption of intestinal signaling interactions—antagonistic effect on the glucagon-like peptide-1 (GLP-1) receptor, a peptide hormone [72]; Increase in serum LPS, increase in intestinal permeability, change in composition of the microbiota [75]; Decrease in total serum antioxidants, increase in serum MDA and NO levels [71]; Infiltration of leukocytes and increase in Kupffer cells [71]; Changes in histological structure [71]; Reduction in Bcl2 expression [71].
Potassium sorbate (E202)/ benzoic acid (E210)/ sodium nitrate (E251)	1 mg/kg body weight/ 5 mg/kg body weight/ 3.7 mg/kg body weight	Qualitative and quantitative change in the composition of the intestinal microbiota changes associated with intestinal epithelial permeability and in the expression of intestinal tight junction markers [82].
Carrageenan (E407)	75 mg/kg body weight	Adverse effects on the intestinal epithelium, composition of the intestinal microbiota and increased expression of pro-inflammatory molecules [90].
Polysorbate 80 (E433)	25 mg/kg body weight	Faster increase in body fat, elevated parameters indicating metabolic syndrome and low-grade inflammation, the presence of abnormalities in the gut microbiota [101].
Maltodextrin (E1400)	-	Increased susceptibility to colitis in genetically susceptible individuals [106]; Modifications in gut microbiota and immune factors [107].
Monosodium glutamate (E621)	30 mg/kg body weight	Infiltration of lymphocytes, macrophages, eosinophils, increasing inflammation which predisposes to hepatocellular carcinoma [117]; Gut dysbiosis [111].
Sucralose (E955)	15 mg/kg body weight	Increased expression of genes related to inflammation and oxidative stress [134]; Reducing the abundance of bacterial communities associated with bile acid metabolism [127]; A reduction in the level of hepatic FXR activation [127]; Disturbance of the composition of the intestinal microbiota; increased production of deoxycholic acid [126]; Impaired glycemic response [128].
Aspartame (E951)	40 mg/kg body weight	Liver dysfunction—increase in liver enzymes (ALT and AST) [134]; Impaired hepatic gluconeogenesis, decreased GSH and GCLc and cysteine levels [137]; Blockage of the trans-sulfuration pathway, increased levels of oxidative stress due to increased MDA and decreased Nrf2 activation [134]; Increased levels of lipid peroxidation [134]; Disturbances in the composition of the microbiota [136].
Saccharin (E954)	9 mg/kg body weight	Increased levels of iNOS and TNF-α in the liver [141]; An increase in the development of inflammation and disturbances in the composition of the intestinal microbiome [141]; Impaired glycemic response [129].

FXR—farnesoid X receptor; ALT—alanine transaminase; AST—aspartate aminotransferase; GSH—glutathione; GCLc—glutamate cysteine ligase; ROS—reactive oxygen species; MDA—malondialdehyde; NO—nitric oxide; LPS—lipopolysaccharide; Nrf2—nuclear factor erythroid 2-related factor 2; iNOS—inducible nitric oxide synthase; TNF-α—tumor necrosis factor alpha.

## Data Availability

Not applicable.
